# Artificial Intelligence for Personalized Genetics and New Drug Development: Benefits and Cautions

**DOI:** 10.3390/bioengineering10050613

**Published:** 2023-05-19

**Authors:** Crescenzio Gallo

**Affiliations:** Department of Clinical and Experimental Medicine, University of Foggia, 71121 Foggia, Italy; crescenzio.gallo@unifg.it

## 1. Introduction

As the global health care system grapples with steadily rising costs, increasing numbers of admissions, and the chronic defection of doctors and nurses from the profession, appropriate measures need to be put in place to reverse this course before it is too late. Fortunately, the technology at our disposal offers cost-effective solutions to these problems. Machine learning (ML) [1,2,3,4,5,6,7,8,9,10,11,12,13,14,15], artificial intelligence (AI) [16,17,18,19,20,21,22,23,24,25,26,27,28,29] and Big Data [30,31,32,33,34,35] can provide robust and innovative answers to long-standing problems.

AI, thanks in part to the many startups engaged in the field, is also facilitating many advancements in pharmaceuticals, helping to reduce research time and costs. The use of artificial intelligence in pharmaceuticals [36,37,38,39,40,41,42,43] has been considerably successful over the past three years, with significant strides being made compared to the previous period, especially when considering its impact on lowering costs and achieving goals in lightning-fast timeframes by the industry standards.

## 2. Predictive Analytics and Personalized Genetics

A very promising technological advancement for the future of health care is predictive analytics [44,45,46,47], used to predict outcomes, events, and behaviors that will or may occur in the future. This technology has the potential to significantly transform health care systems in several countries, creating strong tools to identify and address heath threats, improve patient outcomes, and reduce the cost of medical care. Examining huge quantities of data from a variety of sources, predictive analytics is able to pinpoint models and tendencies that can “spur” the successful deployment of focused actions and programs and assist health care professionals in making better choices about the treatment of their patients. In addition, this technology may assist doctors in developing individualized treatment plans for single patients, helping create better outcomes and lower medical expenses.

Among the application areas of the technology under consideration to the health care sector, personalized genetics represents fertile ground for the use of predictive analytics [48]. We are talking about an approach to health care that uses genetic data, environmental data and data regarding an individual’s lifestyle to devise personalized care plans.

A major advantage of personalized genetics [49] is that it enables health care professionals to identify people who are at increased risk for particular disorders and diseases, such as cancer or heart disease, well in advance of the onset of symptoms. With the early identification of symptoms, health care workers may be able to adopt proactive measures to avoid the progression of a disease, resulting in better outcomes for patients. A further benefit of personalized genetics is that it enables health care professionals to adapt therapies to the particular requirements of each patient according to their individual traits. Thus, for example, as an individual’s genetic data are analyzed, health care practitioners can identify the most effective treatments for that patient and those that, on the other hand, might result in adverse effects. By analyzing an individual’s lifestyle and information about the environment in which he or she lives and performs daily activities, clinicians are able to detect a person’s risk factors and recommend changes in lifestyle that may help avert the occurrence of specific states or diseases.

Predictive analytics employs statistical techniques, ML algorithms and data mining (mining of large amounts of data) both to examine historical data and to precisely forecast upcoming events or results. The technology under consideration entails several processes: data collection and housekeeping, analysis and visualization, feature selection, model creation, and estimation and distribution [50,51]. Data used in predictive analytics applied to health care can come from different sources, including digital medical records and patients’ portable and wearable devices (e.g., smartphones and smartwatches). The fast and very efficient gathering of data is expected to dramatically change the way health care providers process complex information.

Today, it is possible to use the molecular analysis of genomic information to make predictions about patient outcomes, such as the probability that a patient will respond well to a given therapy. Predictive analytics enables clinicians to concentrate on critical and pertinent data sets alone, making accurate and effective care plans for patients. In addition, the technology under consideration can underpin individualized therapy [52] through the identification of genetic factors which render individuals more likely to be prone to specific conditions and by detecting precise genetic markers on which medicines may act. The concept that, going forward, laboratory findings on genomic data may be as simple (and affordable) to obtain as any other blood test cannot be ruled out.

## 3. The Most Significant Achievements of AI in the Pharmaceutical Field

### 3.1. AI against Tumors

A study [53] from the Medizinische Universität Wien (Medical University of Vienna) successfully tested the use of artificial intelligence in the fight against a tumor. In detail, this involved a test carried out on an elderly patient suffering from an aggressive form of blood cancer, which as many as six cycles of chemotherapy had failed to counteract. With each round of “ordinary” treatment, the doctors who had been treating him had crossed off, one after another, all the anticancer drugs that were unsuccessful.

Running out of solutions, doctors had invited the patient to participate in a trial organized by the university, which was in the process of verifying a new kind of research developed by the British company Exscentia [54], accurately tailoring each patient to their required medicines while considering the biological variations between individuals. Researchers in the Austrian study (involving researchers from Tokyo, New York and Zurich) took a small tissue sample from the patient and separated the sample, including normal and cancer cells, into more than a hundred components by exposing each of them to a variety of drug combinations, supported by the use of robotic automation and ML models that were trained to detect small differences in the cells taken from the patient.

The scientists were simply doing what the physicians did: testing several drugs to just get a feel for what worked. Yet, rather than subjecting one patient to several months’ worth of cycles of chemotherapy, the researchers tested dozens of treatments simultaneously, with great success. With the discovery and administration of the right drug, the patient entered into full remission; that is, in fact, his tumor disappeared!

### 3.2. Machine Learning to Design New Drugs

However, selecting the right drug is only part of the problem that Exscientia wants to solve. The company is intent on overhauling the entire drug development chain; in addition to matching patients to existing drugs, it wants to use machine learning to design new ones. The first drugs designed with the help of AI are now in clinical trials; it will be a matter of time until their real levels of effectiveness are verified in various stages of research.

In addition to Exscientia, there are hundreds of startups exploring the use of machine learning in the pharmaceutical industry. Today, on average, it takes more than a decade and billions of dollars to develop a new drug. The “common” goal is to use artificial intelligence to make drug discovery faster, safer and cheaper. By predicting the behavior of potential drugs in the body and discarding compounds that do not work “in advance”, machine learning models can reduce the need for time-consuming and “grueling” laboratory procedures. We are seeing a surge in activity and investment because the increasing automation of the pharmaceutical industry has begun to produce enough chemical and biological data to train effective machine learning models. However, it is still too early to “rest on our laurels”.

## 4. Technology Is Not a Cure-All

There are many companies in the field of artificial intelligence that make assertions they cannot back up, such as, for example, claiming to be able to accurately tell which molecule can pass through the intestine and which molecule cannot be dismembered by the liver. Technology is not a panacea; laboratory cell and tissue experiments and human testing, the most time-consuming and expensive parts of the drug development process, simply cannot be totally eliminated. The use of technology certainly saves much time and money; however, the final validation must always be carried out in the laboratory.

It may be a few more years before the first drugs designed with the help of artificial intelligence reach the market, although, at present, the fundamental phases in new drug development from the ground up have not greatly changed. To begin with, you choose a particular mark in the organism with which the compound is to interact, for example, a protein; next, you engineer a molecule that “does something” for that target, such as modifying its function or turning it off; then, you produce the molecule in the laboratory and make sure it does indeed do just what it was intended for; lastly, it is tested on people to determine if it is secure and effective. In the past few decades, chemists have tested drug candidates by placing samples of the desired target in many small subdivisions in a laboratory, placing several molecules in them, and observing the response. Thus, in this method, such a process is repeated several times, changing the molecular structure of drug candidates and substituting different atoms between them. However, a number of molecules that apparently worked in the lab have ultimately failed when they were tested on individuals.

The new generation of companies in the field of artificial intelligence are concentrating on the following critical points in the process of drug development:

- Identifying the correct drug target in the organism;
- Devising the appropriate molecule for interaction with it;
- Establishing in which populations the molecule has the greatest likelihood of being used successfully.

With machine learning, it is possible to leverage large quantities of data, even drug and molecule data, to automatically create complex models [55]. Doing so makes it much quicker and easier to anticipate the behavior of medicines in an organism, enabling numerous trials to be performed. ML models also can scour large unexploited reservoirs of candidate drug molecules in a manner that was not previously possible [56,57]. This means that the tough, but crucial, laboratory work (and subsequent clinical trial phases) only needs to be carried out on the molecules that are most likely to succeed.

Prior to even getting to the point of simulating drug behavior, several businesses are currently in the process of implementing ML in target identification. Using natural language to extract extensive data from decades-old archives of science reports, among them being hundreds of thousands of published genetic sequences and even millions of papers [58], ML models are able to envisage the most likely targets to concentrate on when attempting to address a particular pathology. However, choosing a specific target is only the beginning. Designing a drug molecule that does “something”, that produces results, is the biggest hurdle. The interplay within the body between molecules is also very intricate. A number of drugs must cross harsh environments, such as the intestines, in order to do their work. The entire process is regulated by both physical and chemical rules working on an atomic scale. Most artificial-intelligence-based drug development efforts aim to “navigate” through the wide range of choices and rapidly identify novel molecules satisfying the greatest possible number of requirements.

## 5. How AI Can Reduce the Time and Cost Associated with New Drug Research?

In any case, beyond anything else, drugs must be tested in humans. Such final steps in drug development, involving the enrollment of huge numbers of participants, are difficult to manage and, in general, time-consuming, sometimes ranging from ten to twenty years. Many drugs “make it;” others fail in clinical trials. Artificial intelligence will not accelerate the process of conducting such trials, yet it might assist pharmaceutical manufacturers in increasing the chances in their favor by reducing the amount of time and expense involved in searching for new drugs. With significantly less time devoted to trialing prospectless drug molecules in the laboratory, potential candidates will advance more quickly to the clinical experimentation stage. Additionally, having fewer funds available, businesses may no longer feel obligated to use a medicine that does not have particularly good results.

Improving patient selection may also contribute to improvements to the whole process [59]. The majority of clinical trials measure a drug’s average effectiveness by counting the number of individuals in whom it was successful and the number in whom is was not. If a sufficient number of people in a given study experience an improvement in their disease, the drug is regarded as a success. If the drug does not work for a sufficient fraction of patients, it is a failure. However, this may also mean that small populations, in whom a particular drug has successfully operated, are disregarded (being part of the “failure”). If the “right patients” could be selected at the source, things might be different. Such is the case with the Austrian study reported in the preface.

The British company Exscentia sampled tissue from dozens of cancer patients subjected to at least two failed rounds of chemotherapy, evaluating the effects of over a hundred existing drugs on their cells. With the collaboration of researchers at the University of Vienna, it succeeded in identifying a treatment that successfully worked for over half of the patients. The company plans to use this technology to model its method of drug development, embedding patient data early in the process to train “improved” artificial intelligence.

## 6. The Impact of Machine Learning Algorithms on Global Health Care

Using predictive analytics, one could find people at risk of being readmitted to hospital, helping health care professionals design tailored actions to lower the chance of continued hospitalizations. Although the technology under consideration is in the early stages, the provision of quick and careful artificial-intelligence-guided data might be very useful in estimating the responsiveness and need for discharging patients “at scale” for future global (and personal, individual patient-level) health emergencies. Predictive analytics can quickly and reliably target high-risk individuals for specific conditions and adapt treatments to particular people based on their individual attributes. Access to life-saving information derived via genomics, employing ML methods to process the vast quantity of data produced via genome sequencing, can be a powerful tool at the disposal of physicians. The technology under consideration can greatly improve the operation of nosocomial hospitals globally by “curbing the leakage” of physicians and nurses, improving data flow and knowledge transfer, and then reducing cost pressures. In addition, it will enforce collaboration efforts between physicians and patients, encouraging trust, clarity, and cost-effectiveness in the future of global health care.

## 7. Conclusions

Predictive analytics holds the promise of redefining health care through the provision of intuition and forecasting that may inspire decision making and enhance patient results. Nevertheless, the challenges associated with using this technology must be addressed to guarantee accurate and reliable results. Dealing with the shortcomings of predictive analytics in health care demands a manifold perspective involving the careful preparation of the data used, the selection of the right models, and the assessment of performance, along with close attention to data protection, safety, and integrity. Although there is great promise for this technology in health care, it is critical to both realize and deal with the difficulties and limitations inherent in its application. In addition, the cumbersome and dynamic profile of health care data demands continuous follow-up and adjustment. Simply put, predictive analytics holds enormous promise to provide significant feasible information for the progress of today’s medicine. Moreover, the earliest batch of medicines designed by AI is still in clinical trials. It may take months, or perhaps years, for the first drugs to pass through and reach the consumer market. A few may fail. However, even if this first group should not succeed, another group will follow. Medicine design, in short, has changed forever.

We will see how the applications of artificial intelligence to medicine evolve in the near future and hopefully help make our world a better place.
